# Free Radical Damage in Ischemia-Reperfusion Injury: An Obstacle in Acute Ischemic Stroke after Revascularization Therapy

**DOI:** 10.1155/2018/3804979

**Published:** 2018-01-31

**Authors:** Ming-Shuo Sun, Hang Jin, Xin Sun, Shuo Huang, Fu-Liang Zhang, Zhen-Ni Guo, Yi Yang

**Affiliations:** ^1^Department of Neurology, The First Hospital of Jilin University, Chang Chun 130021, China; ^2^Clinical Trail and Research Center for Stroke, Department of Neurology, The First Hospital of Jilin University, Chang Chun 130021, China

## Abstract

Acute ischemic stroke is a common cause of morbidity and mortality worldwide. Thrombolysis with recombinant tissue plasminogen activator and endovascular thrombectomy are the main revascularization therapies for acute ischemic stroke. However, ischemia-reperfusion injury after revascularization therapy can result in worsening outcomes. Among all possible pathological mechanisms of ischemia-reperfusion injury, free radical damage (mainly oxidative/nitrosative stress injury) has been found to play a key role in the process. Free radicals lead to protein dysfunction, DNA damage, and lipid peroxidation, resulting in cell death. Additionally, free radical damage has a strong connection with inducing hemorrhagic transformation and cerebral edema, which are the major complications of revascularization therapy, and mainly influencing neurological outcomes due to the disruption of the blood-brain barrier. In order to get a better clinical prognosis, more and more studies focus on the pharmaceutical and nonpharmaceutical neuroprotective therapies against free radical damage. This review discusses the pathological mechanisms of free radicals in ischemia-reperfusion injury and adjunctive neuroprotective therapies combined with revascularization therapy against free radical damage.

## 1. Introduction

Acute ischemic stroke is a leading cause of death and disability worldwide [1–3]. Because of the growth of the older global population, ischemic stroke incidence has increased in recent decades [4]. Acute ischemic stroke contributes to a loss of brain function mainly due to a reduction in cerebral blood flow. Currently, intravenous administration of tissue plasminogen activator (tPA) and endovascular thrombectomy are the two main treatment strategies for acute ischemic stroke [5–7]. Currently, intravenous recombinant tissue plasminogen activator (tPA) is the most effective treatment strategy for acute ischemic stroke and remains the first-choice treatment in clinics worldwide. However, there are limitations in the clinical use of intravenous tPA. First, intravenous administration of tPA must be restricted to a strict time window: within 4.5 hours between the last time the patient exhibited normal behavior and the intravenous treatment. The treatment time window is so narrow that only a small number of patients are eligible for intravenous tPA [8]. Besides, the low successful recanalization rate also influences the rate of a favourable outcome [9]. Additionally, complications such as hemorrhagic transformation and fatal edema are severe and can sometimes aggravate the disease [10].

Successful recanalization of the responsible cerebral vessels which lead to blood reflow is the primary target after the onset of acute ischemic stroke. However, there are also possible complications after revascularization, among which cerebral ischemia-reperfusion injury is one of the most serious. Ischemia-reperfusion injury is a common and inevitable problem after revascularization therapy. Although successful recanalization leads to the restoration of cerebral circulation, a fair amount of patients still do not improve in terms of symptoms and function [11, 12]. Cerebral ischemia-reperfusion injury is defined as a biochemical cascade that causes deteriorative effects in ischemic brain tissue, which compromises and antagonizes the beneficial effect of recanalization [13–15]. During the cerebral ischemia reperfusion phase, the pathophysiological mechanisms include the release of excitotoxic neurotransmitters, intracellular Ca^2+^ accumulation, free radical damage, neuron apoptosis, neuroinflammation, and lipolysis [16–22]. Among these complex pathophysiological mechanisms, free radical damage to the brain plays a pivotal role in the process of ischemia-reperfusion injury during revascularization therapy. In order to get favorable clinical outcomes, there is a clear need to develop adjunctive neuroprotective therapies against ischemia-reperfusion injury. In this review, we focus on the role of two major free radicals (ROS and RNS) in ischemia-reperfusion injury as well as on adjunctive therapies, especially against oxidative/nitrosative stress, that combine revascularization therapy.

## 2. Free Radicals

The brain only makes up 2% of the whole body weight, but represents almost 20% of the body's oxygen consumption, generating more free radicals than other organs. Additionally, brain tissues contain considerable amounts of lipids with unsaturated fatty acids and high concentrations of iron, so the brain is more vulnerable to free radical damage [23]. Free radicals are divided into two main groups: reactive oxygen species (ROS) and reactive nitrogen species (RNS). ROS and RNS play key roles in many pathological processes during ischemia reperfusion. Currently, free radicals' toxicity in ischemia-reperfusion injury is being intensively studied.

### 2.1. Oxidative Stress

#### 2.1.1. ROS and Oxidative Stress

Oxidative stress is caused by excess production of ROS. The major detrimental types of ROS include superoxide anion (O_2_^−^), hydroxyl radicals (OH^−^), and hydrogen peroxide (H_2_O_2_). In the physiological condition, superoxide dismutase (SOD), glutathione peroxidase (GPX), catalase, and other antioxidant enzymes can protect brain tissues against ROS cytotoxicity by catalysis, maintaining a neutral balance [24, 25]. Additionally, ROS play a physiological role in regulating immune system function, maintaining redox homeostasis and participating in many pathways even as secondary messengers [26]. While in the process of cerebral ischemia reperfusion, especially during the reperfusion phase, production of free radicals increases remarkably, leading to the breakdown of antioxidant systems.

**Figure 1:**
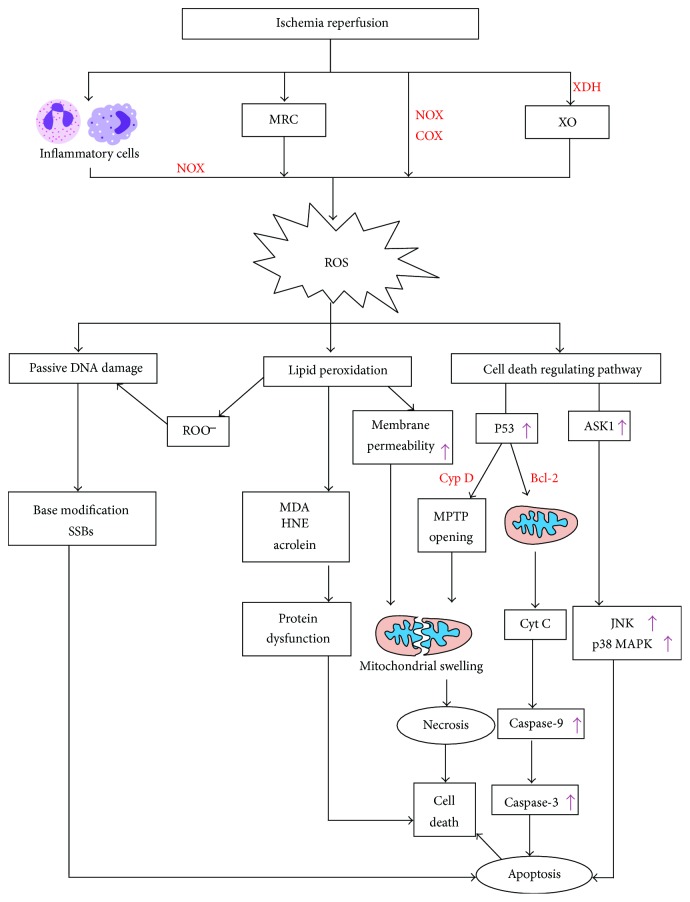
ROS damage in ischemia-reperfusion injury. First, ROS-generated pathways: MRC; NOX; COX-2; XO. ROS react with DNA and then cause passive DNA damage leading to base modification and SSBs which induce to apoptosis. Reaction of OH^−^ with unsaturated fatty acids generates ROO^−^ which may also cause passive DNA damage. The products of lipid peroxidation such as MDA, HNE, and acrolein can lead to protein dysfunction. Besides, lipid peroxidation increases membrane permeability inducing to mitochondrial swelling. P53 activated by ROS can also cause mitochondrial swelling by MPTP opening via reaction of P53 with Cyp D. P53 induces Cyt C released from mitochondria by reacting with Bcl-2 family proteins and subsequently leads to caspase cascade causing apoptosis. What is more, ROS can activate JNK and p38 MAPK pathways which are activated by ASK1 and lead to apoptosis. MRC: mitochondrial respiratory chain; NOX: NADPH oxidases; COX-2: cyclooxygenase-2; XDH: xanthine dehydrogenase; XO: xanthine oxidase; ROS: reactive oxygen species; SSBs: single-strand breaks; ROO^−^: peroxyl radical; MDA: malondialdehyde; HNE: 4-hydroxynonenal; Cyp D: cyclophilin D; Cyt C: cytochrome C; MPTP: mitochondrial permeability transition pore; ASK1: apoptosis signal-regulating kinase 1; JNK: c-Jun NH2-terminal kinase; p38 MAPK: p38 mitogen-activated protein kinase. “↑” demonstrates events that are increased or enhanced.

According to a previous study, excess generation of ROS comes from four pathways: the mitochondrial chain respiratory chain; NADPH oxidases; reaction of arachidonic acid catalyzed by cyclooxygenase 2; and xanthine and hypoxanthine via xanthine oxidase [27] (Figure 1). In the early phase of stroke, ROS are mainly generated from mitochondria. After the onset of ischemic stroke, the brain tissues lack oxygen and glucose, which influences the generation of ATP. With the lack of a supply of ATP, the concentration of calcium in neurons increases, leading to a massive generation of ROS by mitochondrial depolarization [28–30]. Along with macrophage and other immune cell infiltration during neuroinflammation reactions, activation of NADPH oxidase in these immune cells contributes to the generation of ROS, which is called “oxygen burst” when it occurs during reperfusion [31]. NADPH oxidase also produces ROS in other cells, such as vascular endothelial cells. When blood reflows, abundant oxygen arrives, accelerating the oxidative damage. It is well known that oxidative stress can activate proapoptotic signaling like the cytochrome c pathway, inducing DNA damage, changes in protein structure and function, and lipid peroxidation during ischemia and reperfusion [32–35]. Additionally, oxidative stress may directly regulate some important molecules that are found in cellular signaling circuits, such as ion channels and protein kinases [36–39]. In the following section, we will discuss some mechanisms regarding ROS toxicity to brain tissues during ischemia reperfusion.

#### 2.1.2. Oxidative Stress Damage to Brain Tissue in Ischemia-Reperfusion Injury

Oxidative stress can cause cell death via DNA damage, lipid peroxidation, and changes in protein structure and function (Figure 1). DNA damage is divided into two groups: active DNA damage and passive DNA damage, depending on the mechanisms of action. And oxidative stress mainly causes passive DNA damage. Active DNA damage is mediated by DNA endonucleases that mainly contain caspase-activated deoxynuclease, apoptosis-inducing factor, and endonuclease G, which cause DNA double-strand to be fragmented. Passive DNA damage is caused by DNA directly reacting with ROS or DNA indirectly reacting with the products generated from the reaction of ROS and lipids or proteins, leading to modifications of nucleotide bases, such as apurinic/apyrmidinic sites, or formation of single/double-stranded breaks [40]. The hydroxyl radical (OH^−^), a type of ROS that is generated via the Fenton reaction, can lead to lipid peroxidation. OH^−^ reacts with unsaturated fatty acids and produces an alkyl radical, which can form a peroxyl radical (ROOS) in a reaction with molecular oxygen. Then, ROOS receives hydrogen from another fatty acid to produce a lipid hydroperoxide (ROOH) and a second alkyl radical, which starts a cycle of lipid peroxidation [41]. Lipid peroxidation destroys the components of membranes, leading to an increase in permeability, dysfunction of organelles, and changes in ion transport [42]. Additionally, the products of lipid peroxidation play a significant role in oxidative stress injury. These products are a type of reactive aldehyde and include malondialdehyde (MDA), 4-hydroxynonenal (HNE), and acrolein [43–45]. They can lead to the dysfunction of proteins by binding to thiol groups and depletion of GSH through reactions with GSH-Px and glutathione S-transferase, inducing more serious oxidative stress injury.

ROS can also regulate some major apoptosis and necrosis signaling pathways (Figure 1). Protein 53 (p53) is a pivotal molecule in the process of ROS inducing cell death [46, 47]. ROS can activate p53 by reacting with cyclophilin D, which opens the mitochondrial permeability transition pore, leading to mitochondrial swelling [48]. ROS can increase the permeability of the mitochondrial membrane and result in cytochrome c release by forming an inhibitory complex during the reaction of p53 and Bcl-2 family proteins, such as Bax and Bid. Cytochrome c can activate caspases by forming a complex with apoptotic protease activating factor-1, pro-caspase-9, and ATP, which induces apoptosis [49, 50]. P53 upregulated modulator of apoptosis (PUMA), a major proapoptotic protein that is regulated by p53, which belongs to the Bcl-2 protein family [51]. Some researchers have found that inhibiting oxidative stress by abundant SOD1 may suppress PUMA expression, indicating the underlying relationship between oxidative stress and PUMA [47]. Another major pathway regulating cell death, the mitogen-activated protein kinase (MAPK), is also regulated by ROS. It has been reported that MAPK pathways can induce neuronal cell death in the cortex and hippocampus in a transient forebrain ischemia mouse model [52]. The MAPK pathway has three major members: c-Jun NH2-terminal kinase (JNK), extracellular signal-regulated kinase 1/2 (ERK 1/2), and p38 MAPK. JNK and p38 MAPK play key roles in promoting apoptosis, though the function of ERK 1/2 in cell death is controversial. The JNK and p38 MAPK pathways can be activated by apoptosis signal-regulating kinase 1 (ASK1), which is activated by ROS, leading to apoptosis during ischemia reperfusion. Further, JNK's long-lasting activation is ROS dependent [53, 54].

As previously discussed, with the wave of neuroinflammation, immune cells containing NADPH oxidase produce considerable amounts of ROS, aggravating oxidation stress injury. In turn, ROS can also activate these inflammatory cells. ROS activate microglia, neutrophils, and macrophages via the nuclear factor kappa B (NF-*κ*B) pathway. Leucocytes contain myeloperoxidase that can generate hypochloric acid, an intense oxidant, because of the components of Cl^−^ and hydrogen peroxide [55].

### 2.2. Nitrosative Stress

#### 2.2.1. RNS and Nitrosative Stress

Nitrosative stress is mainly caused by RNS. RNS has two major species, NO and ONOO^−^, which mainly participate in the process of ischemia-reperfusion injury. Generally speaking, NO is generated from the enzymatic reaction of L-arginine and oxygen, which is catalyzed by three types of nitric oxide synthase (NOS), including endothelial NOS (eNOS), neuronal NOS (nNOS), and inducible NOS (iNOS). Among these three nitric oxide synthases, eNOS and nNOS are calcium dependent, while iNOS is calcium independent. In most cases, the low concentration of NO produced by eNOS is physiological, whereas NO generated from nNOS and iNOS is harmful. Thus, we can see that NO has two side effects in the brain. The basal concentration of NO, which is less than 10 nmol/L, produced from eNOS plays an essential role in maintaining normal neurocrine, immunological, and vascular physiology [56–61]. Huang et al. found that eNOS knockout mice had larger infarcts than wild-type mice in an ischemic stroke mouse model [62]. Additionally, eNOS may produce a large portion of NO at the early stage of ischemia onset, contributing to mediating vasodilation protectively [63]. Further excess production of NO, primarily generated from activated nNOS and iNOS, is harmful to the ischemic brain [64]. Gursoy et al. suggested that the overactive eNOS may also be harmful and that partial inhibition of eNOS may provide optimum prevention of ischemia-reperfusion injury for the brain by inhibiting peroxynitrite formation [65]. Compared to superoxide dismutase, O_2_^−^ prefers to react with NO and form a strong oxidant peroxynitrite (ONOO^−^), which has much stronger oxidation than NO and O_2_^−^ alone [66].

#### 2.2.2. Nitrosative Stress Damage to Brain Tissue in Ischemia-Reperfusion Injury

Excess NO may lead to the breakdown of the blood-brain barrier (BBB), cell death, and inflammation. The activation of matrix metalloproteinase pathways which is one type of crucial pathways in the opening of the BBB and the distribution of tight junction proteins which are the main components of the BBB are both influenced by NO [63, 65–70]. NO can activate MMP-2 at the first stage of BBB opening and activate MMP-9 at the second stage [71–73]. Activated MMPs degrade the extracellular matrix of the vascular wall and tight junction proteins. Excess NO, especially produced from iNOS and nNOS, can result in cell death via mitochondria dysfunction, pivotal protein modification, and peroxynitrite formation. NO strongly inhibits cytochrome c oxidase in the mitochondria respiratory chain during the ischemia phase [74]. NO can react with protein resulting in nitrosothiol formation or protein nitrosylation [75, 76]. NO, especially generated from iNOS, enhances cyclooxygenase-2 (COX-2) activity, which can mediate glutamate excitotoxicity to produce more ROS and participate in the inflammatory reaction because of proinflammatory prostaglandin E2, the product of the COX-2 reaction [77, 78].

ONOO^−^'s critical damage to the brain consists of cell death and disruption of the BBB. Tyrosine nitration is a major cause of cell death. ONOO^−^ reacts with tyrosine to form 3-nitrotyrosine, leading to dysfunction of some essential proteins because of changes in their structure, such as inhibition of enzymatic activity, cytoskeletal protein disruption, and signal transduction damage [79, 80]. ONOO^−^ reacts with tyrosine through two pathways, including ONOO^−^ reacting with metal ions to produce nitronium ions, which further react with tyrosine residues [81], and tyrosine reacting with the product of the reaction between ONOO^−^ and CO_2_ [82, 83]. Furthermore, ONOO^−^ can react with key elements of DNA, such as guanine nucleotides and the sugar-phosphate backbone, causing DNA damage due to the strong nitration of ONOO^−^, and then activate PARP pathway [66]. PARP-1 is a type of DNA repair enzyme and is activated by DNA damage induced by ONOO^−^. Massive activation of PARP-1 exhausts NAD^+^, leading to cell death [84]. Additionally, ONOO^−^ can also cause dysfunction of the mitochondria by regulating complexes I–V of the mitochondrial respiratory chain [85–89]. Membrane lipid peroxidation caused by ONOO^−^ can also lead to cell death [90]. Except for this critical influence, ONOO^−^ is also associated with MMPs. There are some studies that have found that ONOO^−^ can activate MMP-1, MMP-2, and MMP-9 by different mechanisms [91–93], causing tight junction protein rearrangement and dysfunction, leading to an increase in BBB permeability and disruption of the BBB integrity during ischemia-reperfusion injury.

## 3. Complications after Revascularization Therapy

**Figure 2:**
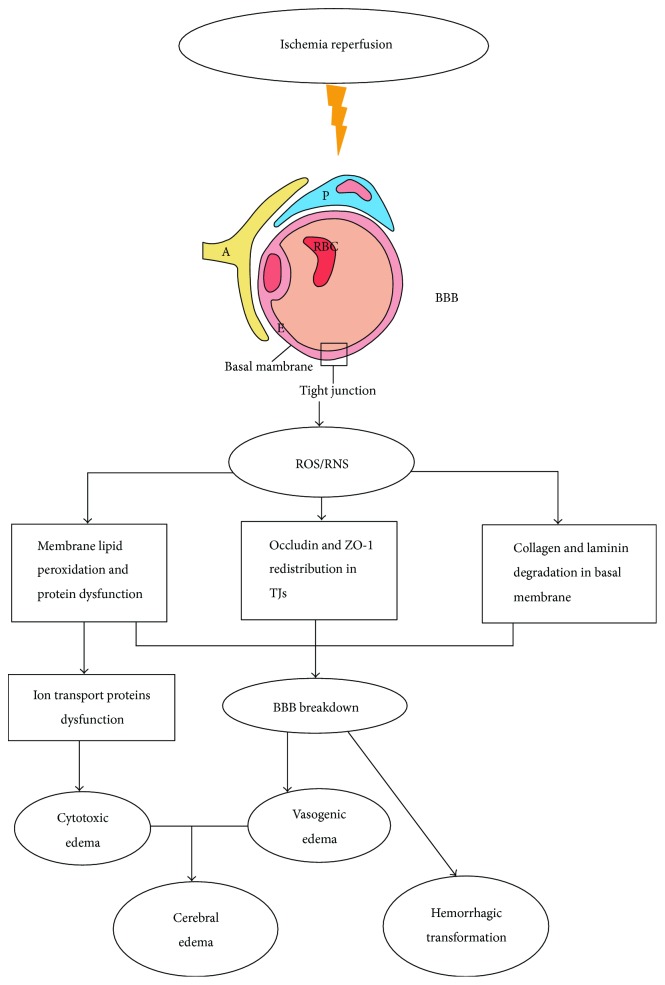
Complications after revascularization therapy. Severe complications include hemorrhagic transformation and cerebral edema. Hemorrhagic transformation is connected with the increase in the permeability of BBB. H_2_O_2_ generated from NADPH oxidase modifies tight junctions by redistribution of occludin and ZO-1. NO/ONOO^−^ degrades collagen and laminin in the basal membrane by the activation of MMP pathways. Free radicals also cause lipid peroxidation and protein dysfunction. All these pathophysiologic processes lead to BBB breakdown and subsequently result in hemorrhagic transformation and vasogenic edema. Cytotoxic edema is associated with the dysfunction of the ion transport in the membranes which suffer lipid peroxidation and protein oxidation. BBB: blood-brain barrier; TJs: tight junctions. The meaning of letter in blood-brain barrier: E: endothelial cells; P: pericytes; A: astrocyte end feet.

Revascularization therapy may cause severe complications, such as hemorrhagic transformation and cerebral edema, and free radical damage during ischemia reperfusion has a strong connection with these complications (Figure 2). Patients who experience these complications may get worse outcomes, even the responsible large vessel recanalization.

### 3.1. Hemorrhagic Transformation

Hemorrhagic transformation is strongly connected with the integrity of the BBB. The BBB is a semipermeable barrier between peripheral circulation and the central nervous system, preventing some blood substances from reaching the brain. The BBB contains vascular endothelial cells, tight junctions, the basal membrane, and pericyte and astrocyte end feet [94] (Figure 2). These cells contain mitochondria and enzymes, such as NAPDH oxidase and NOS [95–98]. During ischemia reperfusion after thrombolysis or thrombectomy, H_2_O_2_ generated from NADPH oxidase can increase BBB permeability through redistribution of occludin and ZO-1, located in tight junctions [99, 100]. Free radicals, especially NO and ONOO^−^, activate the MMP pathways, which lead to collagen and laminin degradation in the basal membrane, inducing breakdown of the BBB [97, 101–106] (Figure 2). Sumii et al. found that MMPs participate in the tPA-associated hemorrhage progress [107], so it is possible that NO participates in hemorrhagic transformation after tPA treatment mediated by the MMP pathway. Additionally, tPA can upregulate MMPs (especially MMP-9) by the lipoprotein receptor signaling pathway and can reduce hemorrhagic volumes by using MMP inhibitors [68, 107–110], but the effect of the combination of NO and tPA on hemorrhagic transformation is still unknown. Further, many researchers have found that ONOO^−^ can decrease tPA activity, influencing the process of thrombosis and thrombolysis [111–113].

### 3.2. Cerebral Edema

Edema caused by free radicals can be divided into vasogenic edema and cytotoxic edema. Vasogenic edema is related to the increased permeability of the BBB, the mechanisms of which are discussed above. Cytotoxic edema is connected with the dysfunction of ion transport in membranes. The ion transport proteins oxidized by ROS include ion channels, ion pumps, ion exchangers, and ion cotransporters. ROS can peroxidize membrane phospholipids, oxidize sulfhydryl groups located on the ion transport proteins, inhibit oxidative phosphorylation, and decrease ATP levels, which will induce dysfunction of ion transport leading to cytotoxic edema [114, 115] (Figure 2). Additionally, ROS participate in inhibition of the uptake of glutamate by Na^+^/glutamate transport. Massive glutamate released during ischemia reperfusion destroys the homeostasis of Na^+^, K^+^, and Ca^2+^, leading to dysfunction of membranes and cytotoxic edema [116].

## 4. The Neuroprotective Therapies Combined with Revascularization Therapy

Free radical damage in ischemia-reperfusion injury severely impacts prognosis after revascularization therapy. Thus, determining how to protect the brain from free radical damage is extremely urgent. In the following section, we review some of the most recent and effective therapies used to prevent free radical damage in ischemia-reperfusion injury that are used in combination with revascularization therapy. The adjunctive strategies have been both understudied in experimental and clinical research. The therapies are divided into two groups: nonpharmaceutical and pharmaceutical. Nonpharmaceutical therapies consist of remote ischemic conditioning and hypothermia, and pharmaceutical therapies consist of edaravone, uric acid, and citicoline.

### 4.1. Nonpharmaceutical Combined with Revascularization Therapy

#### 4.1.1. Remote Ischemic Conditioning

Remote ischemic conditioning (RIC) was first introduced in a canine cardiac study by Przyklenk et al. [117]. According to the initiation time of the transient period of ischemia, RIC is divided into three types: RIPC, RIPerC, and RIPostC. (1) RIPC is a strategy of conducting a transient, precedent ischemic stimulus far from the target organ or tissue to protect the target against subsequent, more prolonged, and severe ischemia or ischemia-reperfusion injury [117, 118]. (2) RIPerC is a procedure in which RIC is conducted during ischemia but before reperfusion of the target organ [119, 120]. Compared with RIPC, RIPerC is more suitable for clinical events because stroke is an emergency situation during which RIPC cannot be performed properly. Further, according to Hahn's study, RIPerC is more effective than RIPC in protecting the brain against ischemia-reperfusion injury [121]. (3) RIPostC is a strategy that involves starting RIC after the ischemic period of the target organ, but during reperfusion [122]. After many years of research, limbs have been found to be the most suitable remote conditioned sites because of the effectiveness, feasibility, and safety [123–125]. Animal experiments primarily choose hind limbs [123, 125–127], while clinical studies prefer to use upper limbs as the remote conditioned site [124, 128].

**Figure 3:**
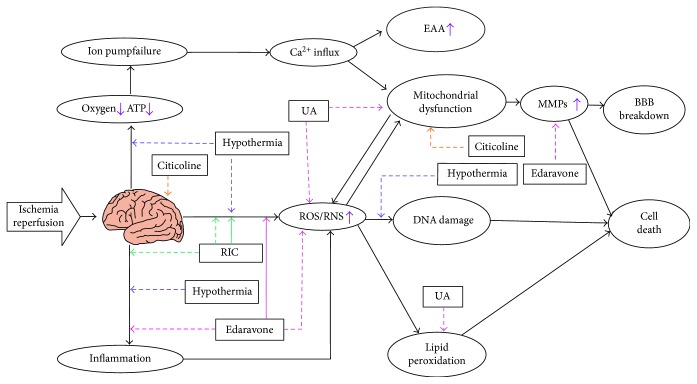
Adjunctive therapies against free radical damage in ischemia-reperfusion injury. RIC improves cerebral blood flow by increase in the generation of NO and inhibits the activation of NADPH oxidase in neutrophils. Hypothermia decreases the generation of free radicals, inhibits the induction of oxidative DNA lesions, and suppresses immune system. Edaravone eliminates free radicals and enhances the release of NO. Besides, edaravone also inhibits inflammatory responses and MMP cascade. UA can scavenge free radicals and suppress lipid peroxidation. Citicoline plays a key role in maintenance of membrane against free radical damage. Dashed line indicates inhibition, whereas real line indicates enhancement. UA: uric acid; RIC: remote ischemic conditioning; EAA: excitatory amino acid. “↑” demonstrates events that are increased or enhanced.

The mechanisms of protection from ischemia-reperfusion injury are under discussion, and some specific mechanisms remain unknown (Figure 3). Pan et al. concluded that there are three pathways that explain how RIC protects the brain from ischemia-reperfusion injury: the humoral pathway, immunological pathway, and neuronal pathway [129]. In the humoral pathway, many humoral factors, such as NOS, erythropoietin, heme oxygenase-1, angiotensin-1, and adenosine A1 receptor, have been found in ischemic stroke studies [130–132]. Pignataro et al. found that expression of nNOS in the central nervous system is elevated by RIPostC in MCAO rats. Although nNOS is harmful during ischemia reperfusion as discussed above, some studies have already determined its role in protection in RIC by inhibition of oxidative/nitrosative stress [132–134]. In addition to nNOS, eNOS upregulated by the PI3K/Akt pathway also takes part in neuroprotection in RIC [135]. These NO may improve cerebral blood flow after ischemia onset. RIPostC can also inhibit the activation of NADPH oxidase in neutrophils in a rat MCAO model [136]. Additionally, the immunological pathway leads to inhibition of systemic inflammation [137], and the neuronal pathway protects the brain mainly via participation of the vagus nerve combined with the humoral pathway [127, 138, 139].

Although most of the evidence of protection by RIC is from cardiac research, an increasing number of studies have indicated that RIC is effective in protecting the brain against ischemia-reperfusion injury. As mentioned previously, RIPerC and RIPostC are more suitable for clinical events. Hoda et al. used a murine embolic clot model of stroke, which is better able to physiologically and clinically represent human stroke cases, and found that RIPerC performed 2 h after MCAO combined with alteplase injected 4 h after MCAO was more effective than RIPerC or alteplase alone [126]. In 2014, Hougaard et al. conducted a randomized clinical trial to show that RIPerC may be neuroprotective. Hougaard et al. collected acute ischemic stroke patients within the 4.5 h thrombolysis time window and randomly conducted prehospital RIPerC at a 1 : 1 ratio when patients were transferred to the hospital in an ambulance. When patients arrived at the hospital, alteplase treatment was given immediately after MRI scanning and National Institutes of Health Stroke Scale scoring. Although penumbral salvage, final infarct size, infarct growth, and clinical outcome did not differ between groups, voxelwise analysis showed that prehospital RIPerC reduced the tissue risk of the infarction and RIPerC had neuroprotective effects [128]. Thus, we can see that although RIC is still new, it represents a promising treatment, and more clinical research regarding this technique is needed.

#### 4.1.2. Therapeutic Hypothermia

Therapeutic hypothermia as a method of neuroprotection against acute ischemic stroke has recently seen extensive research. Therapeutic hypothermia in ischemic stroke involves methods to reduce temperature (especially brain temperature) to a specific range during or even after ischemia to protect neurons in the brain. Methods to induce hypothermia include whole body surface cooling or selective head cooling as well as whole body intravenous cooling or intra-arterial local cooling, which may or may not be combined with hypothermia-inducing drugs. Each method has advantages and disadvantages, and there is no conclusion regarding which technique is best [140]. Generally speaking, therapeutic hypothermia is described according to the target temperature as mild, moderate, and severe, corresponding to 35°C–32°C, 32°C–25°C, and <25°C, respectively [141]. Although therapeutic hypothermia has been widely researched and detailed in cardiac aspects, the best methods and target temperature in acute ischemic stroke are still under discussion.

The mechanism of hypothermia against ischemia-reperfusion injury has been recently studied (Figure 3). Hypothermia places the body in a low metabolic situation, in which most physiological and pathophysiology activities are at low levels. The body requires less oxygen and ATP; thus, the brain can conserve energy, decreasing the generation of free radicals, lactate, and excitatory amino acids [142, 143]. Many studies have documented that hypothermia can inhibit the generation of free radicals against oxidative/nitrosative stress during ischemia reperfusion, further protecting brain cells [144–148]. Ji et al. also found that mild selective brain hypothermia during the ischemia phase can reduce the volume of infarction, suppress induction of oxidative DNA lesions, and attenuate prodeath signaling pathways in a rat MCAO model [148]. Further, the immune system response and apoptosis are also suppressed by hypothermia [149–155]. Additionally, hypothermia can also prevent brain edema and hemorrhage by protecting the BBB and suppressing aquaporin 4 expression [156, 157].

Some studies have found that cooling experiments can decrease the activity of tPA [141, 158]. However, therapeutic hypothermia combined with revascularization therapy has been studied, and a majority of the results indicate that therapeutic hypothermia is safe and feasible, even in endovascular treatment, despite many results regarding better prognosis being neutral. Kallmunzer et al. found mild hypothermia combined with tPA treatment may reduce infarct volume and prevent the disruption of the BBB in a thromboembolic stroke model, indicating that mild hypothermia has neuroprotective function against ischemia-reperfusion injury [159]. Studies in a rat embolic stroke model showed that mild hypothermia may extend tPA therapeutic time window up to 6 hours [160]. However, Kollmar et al. found no significant differences in the survival rate and final infarct volume between IV tPA combined with mild hypothermia with IV tPA alone in a rat thromboembolic occlusion model [161]. In terms of clinical trials, a randomized, multicenter trial (ICTuS-L) found that mild IV tPA combined with hypothermia by endovascular medication using meperidine and buspirone after whole infusion of tPA had no benefit in terms of favorable outcomes at 90 days compared to IV tPA alone, and pneumonia was found more frequently in the hypothermia group [162]. A similar result was also found in a single-blind, single-center study [163]. Although a randomized controlled trial found fewer serious adverse events than the ICTuS-L, adverse events, such as hypoxemia, hypercapnia, and acidosis in blood gas analysis as well as shivering, hypomagnesemia, and hyponatremia, were higher than in the normothermia group. Additionally, the results in terms of a good outcome (modified Rankin Scale, 0–2) at 3 months were the same. If a poor outcome was defined as mRS = 3 to 6, unlike the definition used in the article of mRS = 4 to 6, the results for a poor outcome also exhibited no difference [164]. Although the previously discussed results are not optimistic, a prospective cohort study found an inspiring result. It was found that mild hypothermia could reduce the risk of cerebral edema and hemorrhagic transformation and had a better outcome in patients with anterior circulation occlusion that was successfully recanalized by IV tPA or endovascular treatment before conducting mild hypothermia, indicating the protection of hypothermia against ischemia-reperfusion injury [165]. A similar conclusion was also drawn from a 30-patient study [166]. The primary difference between these studies described above is that the patients participating in Hong's and Ma's study all exhibited anterior circulation occlusion and had successful recanalization. Hypothermia protects the BBB by inhibiting the generation of free radicals when vast oxygen reflow along with blood during reperfusion after successful recanalization, further reducing cerebral edema and brain hemorrhage. Thus, hypothermia may be more effective in patients with blood reflow. The hypothermia group patients in the ICTuS-L study were hospitalized in the intensive care units for at least 36 hours after treatment and may have received more attention, causing an ascertainment bias. Although most research has presented neutral results, the better outcomes that were found during reperfusion are still promising, especially after successful recanalization. Its safety and feasibility may allow hypothermia to be used more frequently or to even lengthen the thrombolysis therapeutic window. Additionally, almost all clinical researches regarding hypothermia combined with intravenous thrombolysis or endovascular treatment have been mild (35°C–32°C), so other ranges of target temperatures should be the focus of further research. In brief, the protection against ischemia-reperfusion injury during recanalization may allow therapeutic hypothermia to be more promising and useful.

### 4.2. Pharmaceutical Combined with Revascularization Therapy

#### 4.2.1. Edaravone

Edaravone, a free radical scavenger, has been widely used as an acute stroke treatment in Japan and China for a long period of time. Edaravone can eliminate free radicals during ischemia reperfusion and has inhibitory effects on lipid peroxidation in the arachidonate cascade. Edaravone can also inhibit inflammatory responses during ischemia reperfusion [167–169] (Figure 3). In an animal experiment, edaravone enhanced the release of NO generated from eNOS, but not from iNOS, which can play a protective role in the brain [170]. Further, edaravone can inhibit matrix metalloproteinase-9 (MMP-9) expression in the ischemic brain and protect BBB integrity [171]. Edaravone functions by attenuating cerebral edema, reducing hemorrhagic transformation, and leading to neuroprotection. Because of its numerous advantages, many researchers have focused on edaravone connected with thrombolysis.

Proteolytic activity is one of the deleterious effects of tPA, causing extravasation of tPA from the cerebral vessels, which leads to neuronal damage. In a thromboembolic stroke model, Kano et al. found that edaravone injection combined with tPA concomitantly could protect reperfused cerebral vessels and significantly attenuate extravasation, probably by prevention of peroxidation of vascular endothelial cells [172]. Hemorrhagic transformation after thrombolytic therapy is a severe complication, and edaravone can also play a role in reduction of this symptom. Yagi et al. found that edaravone can inhibit tPA-induced cerebral hemorrhagic transformation by inhibiting MMP-9 expression, both in vivo and in vitro [171]. Further, Yamashita et al. found that edaravone can accelerate the thrombolysis speed induced by tPA in animal models, indicating that edaravone may be useful in thrombolysis with tPA in clinics [173]. Recently, many studies have illustrated that edaravone treatment combined with thrombolysis with tPA is useful and feasible in clinical trials, even in patients older than 80 years. In a multicenter, single-blind, randomized, open-label study, patients injected with edaravone combined with tPA simultaneously had more early recanalization rates within 60 minutes after tPA infusion and better neurological recovery at 24 hours, in comparison with nonedaravone patients [174]. A Japanese research showed that edaravone combined with tPA may improve early outcomes with a lower mRS scores at discharge [175]. Additionally, PROTECT4.5, a prospective observational study in which edaravone was injected intravenously twice a day (in the morning and late afternoon) for several days and tPA was administered at a dose of 0.6 mg/kg, had better outcomes at 3 months in patients with a NIHSS score ≥ 16 and numerically lower incidence of symptomatic intracranial hemorrhage within 36 hours compared to SITS-ISTR [176]. The same conclusion was also drawn in a retrospective study including 129 consecutive patients [177]. Although this conclusion is gratifying, PROTECT4.5 is an observational and nonrandomized study and the dose of tPA that was administered was 0.6 mg/kg. Nevertheless, cotreatment of edaravone and tPA is fairly promising and has provided satisfying results in many studies. To apply these findings to clinical practice, a large-scale randomized clinical trial will need to be undertaken in the future.

#### 4.2.2. Uric Acid

Uric acid (UA) is the end oxidative product of purine nucleotides catalyzed by xanthine oxidoreductase, an endogenous free radical scavenger. UA can scavenge free radicals, such as hydroxyl radicals, hydrogen peroxide, and ONOO^−^. Additionally, UA can inhibit the Fenton reaction, prevent mitochondrial damage, and suppress lipid peroxidation [178] (Figure 3). Further, UA plays a key role in increasing the activity of SOD3 via preventing the inhibition by H_2_O_2_ under a vascular oxidant stress condition [179].

Presently, many researchers have found that UA has a neuroprotective function, including those treated with thrombolysis, due to its antioxidant properties. The high serum uric acid at admission or obtained within a median 24 hours after stroke onset was independently associated with better clinical outcome at 7 days or 90 days in tPA patients [180–182]. However, Lee et al. found that higher tertile serum UA levels (≥5.3 mg/dl) drawn in the emergency room were associated with excellent functional outcomes (according to responder analysis) only in patients with severe baseline deficits whose NIHSS score was ≥15, and the association was found in males, but not in females [183]. The different conclusions between these studies may be due to the different statistical methods and definitions of the endpoint event. UA exhibits such strong antioxidation that some studies have focused on whether exogenous UA may provide protection in our brain. A rat experiment revealed that UA injected intravenously combined with tPA reduced the infarct volume and resulted in better neurologic function than using tPA alone [184]. There are also some clinical studies that have obtained good results regarding the use of UA. In a multicenter, randomized, placebo-controlled, double-blind, phase 2b/3 trial, UA treatment combined with intravenous thrombolysis followed by thrombectomy was found to be safe and improve stroke outcomes compared to a placebo group, although the disability level at 90 days did not differ between the two groups [185].

There is an interesting phenomenon that should be addressed: UA exhibits different effects depending on gender. Llull et al. found that cotreatment of UA and tPA could reduce infarct growth and produce better outcomes than the placebo group only in women, not in men [186]. The gender difference was also present in the prognosis of the clinical outcome at 90 days. Zhang et al. divided people into four groups according to UA levels. The highest quartile of UA level was beneficial for good functional outcomes in females, while the second quartile of UA levels was an independent predictor of better outcomes in males. Patients with the third quartile of UA level were connected with the worst functional outcomes in females, while the highest UA level had the worst outcomes in males [187]. The mechanisms of this gender difference are still unknown, but may be due to hormone levels and different pathways. Premenopausal women have an “estrogen defense” that may influence UA levels. Additionally, women have lower physiological levels of UA and a lower UA-mediated antioxidant capacity, so they may need antioxidants more urgently [188]. Considerable experimental data has indicated that there are differences in ischemia-induced cell death pathways between males and females. Liu et al. found that females can benefit from caspase inhibition, but not males [189]. These explanations may illustrate the gender difference, but a firm conclusion has not been drawn. More research should be conducted regarding these gender differences, and more clinical trials should be conducted.

#### 4.2.3. Citicoline

Citicoline is an essential exogenous form of cytidine-50-diphosphocholine that is involved in the formation of phosphatidylcholine and is also very important for maintenance of the membrane [190–194]. Ischemia-reperfusion injury causes cell membranes to be broken down and decreases the synthesis of structural phospholipids. When the brain is under ischemia attack, membrane phospholipids will be degraded into free fatty acids by phospholipase A2 (PLA2) and free fatty acids will promote the formation of free radicals [195] (Figure 3). Citicoline may inhibit the activation of PLA2 and reduce the release of arachidonic acid. Citicoline plays a key role in stabilizing and repairing membranes, which can preserve the natural defense of cells against oxidative damage [190, 196–199]. Additionally, citicoline also favors the synthesis of nucleic acids and proteins, especially at the nuclear and mitochondrial levels [191, 193]. Citicoline can make a critical difference in the maintenance of cellular and subcellular functions, such as restoring the activity of mitochondrial ATPAse and membrane Na^+^/K^+^ ATPAse, maintaining ion homeostasis, inhibiting the generation of free radicals, and promoting the reabsorption of cerebral edema [200, 201]. Citicoline can also protect the BBB after ischemia followed by reperfusion [202] and has antiapoptotic effects [203–206]. Citicoline may have already been shown to be beneficial at different levels of the ischemic cascade and for inhibiting reperfusion injury against free radical damage in both experimental and clinical studies [207–209].

Andersen et al. reported that a lower dose citicoline combined with tPA reduced the size of brain infarcts more than using tPA alone [210]. María et al. performed a rat experiment that used an embolic stroke model that involved treatment of tPA when combined with citicoline. The sample was divided into 5 groups: control; only using tPA after embolization; only using citicoline after embolization; first using citicoline after embolization combined with subsequently intravenous infusion of tPA; and first using citicoline after thrombolysis by injecting tPA intravenously 10 minutes later. According to the rats' mortality, neurological score, volume of the ischemic lesion, and neuronal death after 72 hours, the use of citicoline after intravenous infusion of tPA achieved the best effect; otherwise, using citicoline before thrombolysis was the same as using tPA alone, indicating that citicoline plays a key role in neuroprotection to inhibit ischemia-reperfusion injury after revascularization [211]. Although animal experiments have exhibited good results, clinical trials have not provided a satisfactory conclusion. In a randomized, placebo-controlled, sequential trial, Dávalos et al. concluded that citicoline had neutral effects in the treatment of moderate to severe acute ischemic stroke, even in a subgroup analysis of patients who received tPA [212]. However, there are many explanations for these neutral results such as the high stroke care standard, which can benefit recovery especially in nonthrombolysis patients, and the higher average NIHSS value in Davalos' study. Although a number of studies support citicoline combined with tPA as being beneficial for protecting the brain from ischemia-reperfusion injury, there are still some disappointing results, especially in clinical trials, and more research should be conducted in this area.

## 5. Conclusions

Free radicals, especially ROS and RNS, have intense oxidation or nitrification abilities in the human brain. During cerebral ischemia reperfusion, especially with blood reflow, massive generation of ROS and RNS leads to cell death via DNA damage, protein dysfunction, and lipid peroxidization. Oxidative/nitrosative stress in ischemia-reperfusion injury also plays a key role in inducing hemorrhagic transformation and cerebral edema after revascularization therapy. Fortunately, many basic experiments and clinical trials have indicated that cotreatment of antifree radical damage strategies, including nonpharmaceutical therapies, such as RIC and hypothermia, and pharmaceutical therapies, such as edaravone, UA, and citicoline, with revascularization therapy is safe and feasible. Further research regarding therapies that prevent free radical damage in ischemia-reperfusion injury combined with revascularization therapy should be undertaken in the future. We will understand the pathomechanisms more deeply, and patients will get better benefits from revascularization therapy.

## References

[1] Jauch E. C., Saver J. L., Adams H. P. (2013). Guidelines for the early management of patients with acute ischemic stroke: a guideline for healthcare professionals from the American Heart Association/American Stroke Association. *Stroke*.

[2] Towfighi A., Saver J. L. (2011). Stroke declines from third to fourth leading cause of death in the United States: historical perspective and challenges ahead. *Stroke*.

[3] Feigin V. L., Forouzanfar M. H., Krishnamurthi R. (2014). Global and regional burden of stroke during 1990-2010: findings from the global burden of disease study 2010. *The Lancet*.

[4] Ovbiagele B., Goldstein L. B., Higashida R. T. (2013). Forecasting the future of stroke in the United States: a policy statement from the American Heart Association and American Stroke Association. *Stroke*.

[5] Röther J., Ford G. A., Thijs V. N. S. (2013). Thrombolytics in acute ischaemic stroke: historical perspective and future opportunities. *Cerebrovascular Diseases*.

[6] Wardlaw J. M., Murray V., Berge E., del Zoppo G. J. (2014). Thrombolysis for acute ischaemic stroke. *The Cochrane Database of Systematic Reviews*.

[7] Goyal M., Menon B. K., van Zwam W. H. (2016). Endovascular thrombectomy after large-vessel ischaemic stroke: a meta-analysis of individual patient data from five randomised trials. *The Lancet*.

[8] Kleindorfer D., Kissela B., Schneider A. (2004). Eligibility for recombinant tissue plasminogen activator in acute ischemic stroke: a population-based study. *Stroke*.

[9] Leiva-Salinas C., Patrie J. T., Xin W., Michel P., Jovin T., Wintermark M. (2016). Prediction of early arterial recanalization and tissue fate in the selection of patients with the greatest potential to benefit from intravenous tissue-type plasminogen activator. *Stroke*.

[10] Annan M., Gaudron M., Cottier J. P. (2015). Functional outcome of hemorrhagic transformation after thrombolysis for ischemic stroke: a prospective study. *Cerebrovascular Diseases Extra*.

[11] Dalkara T., Arsava E. M. (2012). Can restoring incomplete microcirculatory reperfusion improve stroke outcome after thrombolysis?. *Journal of Cerebral Blood Flow & Metabolism*.

[12] Molina C. A., Saver J. L. (2005). Extending reperfusion therapy for acute ischemic stroke: emerging pharmacological, mechanical, and imaging strategies. *Stroke*.

[13] Yang G. Y., Betz A. L. (1994). Reperfusion-induced injury to the blood-brain barrier after middle cerebral artery occlusion in rats. *Stroke*.

[14] Aronowski J., Strong R., Grotta J. C. (1997). Reperfusion injury: demonstration of brain damage produced by reperfusion after transient focal ischemia in rats. *Journal of Cerebral Blood Flow & Metabolism*.

[15] Pan J., Konstas A. A., Bateman B., Ortolano G. A., Pile-Spellman J. (2007). Reperfusion injury following cerebral ischemia: pathophysiology, MR imaging, and potential therapies. *Neuroradiology*.

[16] Cooper H. K., Zalewska T., Kawakami S., Hossmann K. A., Kleihues P. (1977). The effect of ischaemia and recirculation on protein synthesis in the rat brain. *Journal of Neurochemistry*.

[17] Chan P. H., Kerlan R., Fishman R. A. (1983). Reductions of Γ-aminobutyric acid and glutamate uptake and (Na^+^+ K^+^)-ATPase activity in brain slices and Synaptosomes by arachidonic acid. *Journal of Neurochemistry*.

[18] Braughler J. M. (1985). Lipid peroxidation-induced inhibition of γ-aminobutyric acid uptake in rat brain synaptosomes: protection by glucocorticoids. *Journal of Neurochemistry*.

[19] Krause G. S., White B. C., Aust S. D., Nayini N. R., Kumar K. (1988). Brain cell death following ischemia and reperfusion: a proposed biochemical sequence. *Critical Care Medicine*.

[20] Sato M., Hashimoto H., Kosaka F. (1990). Histological changes of neuronal damage in vegetative dogs induced by 18 minutes of complete global brain ischemia: two-phase damage of Purkinje cells and hippocampal CA1 pyramidal cells. *Acta Neuropathologica*.

[21] Siesjo B. K. (1992). Pathophysiology and treatment of focal cerebral ischemia. Part I: pathophysiology. *Journal of Neurosurgery*.

[22] Wang X., Lo E. H. (2003). Triggers and mediators of hemorrhagic transformation in cerebral ischemia. *Molecular Neurobiology*.

[23] Dringen R. (2000). Metabolism and functions of glutathione in brain. *Progress in Neurobiology*.

[24] Yoshioka M., Tanaka K.-i., Miyazaki I. (2002). The dopamine agonist cabergoline provides neuroprotection by activation of the glutathione system and scavenging free radicals. *Neuroscience Research*.

[25] Robinson N. J., Winge D. R. (2010). Copper metallochaperones. *Annual Review of Biochemistry*.

[26] Valko M., Leibfritz D., Moncol J., Cronin M. T. D., Mazur M., Telser J. (2007). Free radicals and antioxidants in normal physiological functions and human disease. *The International Journal of Biochemistry & Cell Biology*.

[27] Margaill I., Plotkine M., Lerouet D. (2005). Antioxidant strategies in the treatment of stroke. *Free Radical Biology & Medicine*.

[28] Drose S., Brandt U. (2012). Molecular mechanisms of superoxide production by the mitochondrial respiratory chain. *Advances in Experimental Medicine and Biology*.

[29] Kalogeris T., Bao Y., Korthuis R. J. (2014). Mitochondrial reactive oxygen species: a double edged sword in ischemia/reperfusion vs preconditioning. *Redox Biology*.

[30] Shenoda B. (2015). The role of Na^+^/Ca^2+^ exchanger subtypes in neuronal ischemic injury. *Translational Stroke Research*.

[31] Walder C. E., Green S. P., Darbonne W. C. (1997). Ischemic stroke injury is reduced in mice lacking a functional NADPH oxidase. *Stroke*.

[32] Nelson C. W., Wei E. P., Povlishock J. T., Kontos H. A., Moskowitz M. A. (1992). Oxygen radicals in cerebral ischemia. *American Journal of Physiology-Heart and Circulatory Physiology*.

[33] Lee S. J., Cho K. S., Koh J. Y. (2009). Oxidative injury triggers autophagy in astrocytes: the role of endogenous zinc. *Glia*.

[34] Lee S. J., Koh J. Y. (2010). Roles of zinc and metallothionein-3 in oxidative stress-induced lysosomal dysfunction, cell death, and autophagy in neurons and astrocytes. *Molecular Brain*.

[35] Granzotto A., Sensi S. L. (2015). Intracellular zinc is a critical intermediate in the excitotoxic cascade. *Neurobiology of Disease*.

[36] Takahashi N., Kozai D., Kobayashi R., Ebert M., Mori Y. (2011). Roles of TRPM2 in oxidative stress. *Cell Calcium*.

[37] Luczak E. D., Anderson M. E. (2014). CaMKII oxidative activation and the pathogenesis of cardiac disease. *Journal of Molecular and Cellular Cardiology*.

[38] Veit F., Pak O., Brandes R. P., Weissmann N. (2015). Hypoxia-dependent reactive oxygen species signaling in the pulmonary circulation: focus on ion channels. *Antioxidants & Redox Signaling*.

[39] Wang F., Reece E. A., Yang P. (2015). Advances in revealing the molecular targets downstream of oxidative stress–induced proapoptotic kinase signaling in diabetic embryopathy. *American Journal of Obstetrics & Gynecology*.

[40] Li P., Hu X., Gan Y., Gao Y., Liang W., Chen J. (2011). Mechanistic insight into DNA damage and repair in ischemic stroke: exploiting the base excision repair pathway as a model of neuroprotection. *Antioxidants & Redox Signaling*.

[41] Beal M. F. (1996). Mitochondria, free radicals, and neurodegeneration. *Current Opinion in Neurobiology*.

[42] Nigam S., Schewe T. (2000). Phospholipase A_2_s and lipid peroxidation. *Biochimica et Biophysica Acta (BBA) - Molecular and Cell Biology of Lipids*.

[43] Esterbauer H., Schaur R. J., Zollner H. (1991). Chemistry and biochemistry of 4-hydroxynonenal, malonaldehyde and related aldehydes. *Free Radical Biology & Medicine*.

[44] Parola M., Bellomo G., Robino G., Barrera G., Dianzani M. U. (1999). 4-Hydroxynonenal as a biological signal: molecular basis and pathophysiological implications. *Antioxidants & Redox Signaling*.

[45] Uchida K. (1999). Current status of acrolein as a lipid peroxidation product. *Trends in Cardiovascular Medicine*.

[46] Saito A., Hayashi T., Okuno S., Nishi T., Chan P. H. (2005). Modulation of p53 degradation via MDM2-mediated ubiquitylation and the ubiquitin–proteasome system during reperfusion after stroke: role of oxidative stress. *Journal of Cerebral Blood Flow & Metabolism*.

[47] Niizuma K., Endo H., Nito C., Myer D. J., Chan P. H. (2009). Potential role of PUMA in delayed death of hippocampal CA1 neurons after transient global cerebral ischemia. *Stroke*.

[48] Vaseva A. V., Marchenko N. D., Ji K., Tsirka S. E., Holzmann S., Moll U. M. (2012). p53 opens the mitochondrial permeability transition pore to trigger necrosis. *Cell*.

[49] Endo H., Kamada H., Nito C., Nishi T., Chan P. H. (2006). Mitochondrial translocation of p53 mediates release of cytochrome c and hippocampal CA1 neuronal death after transient global cerebral ischemia in rats. *Journal of Neuroscience*.

[50] Gomez-Lazaro M., Galindo M. F., Melero-Fernandez de Mera R. M. (2007). Reactive oxygen species and p38 mitogen-activated protein kinase activate Bax to induce mitochondrial cytochrome c release and apoptosis in response to malonate. *Molecular Pharmacology*.

[51] Shan Z., Liu Q., Li Y., Wu J., Sun D., Gao Z. (2017). PUMA decreases the growth of prostate cancer PC-3 cells independent of p53. *Oncology Letters*.

[52] Takagi Y., Nozaki K., Sugino T., Hattori I., Hashimoto N. (2000). Phosphorylation of c-Jun NH_2_-terminal kinase and p38 mitogen-activated protein kinase after transient forebrain ischemia in mice. *Neuroscience Letters*.

[53] Davis R. J. (2000). Signal transduction by the JNK group of MAP kinases. *Cell*.

[54] Song J., Cho K. J., Cheon S. Y. (2013). Apoptosis signal-regulating kinase 1 (ASK1) is linked to neural stem cell differentiation after ischemic brain injury. *Experimental & Molecular Medicine*.

[55] Mattson M. P. (2005). NF-κB in the survival and plasticity of neurons. *Neurochemical Research*.

[56] Moncada S., Palmer R. M., Higgs E. A. (1991). Nitric oxide: physiology, pathophysiology, and pharmacology. *Pharmacological Reviews*.

[57] Margaill I., Allix M., Boulu R. G., Plotkine M. (1997). Dose- and time-dependence of L-NAME neuroprotection in transient focal cerebral ischaemia in rats. *British Journal of Pharmacology*.

[58] Kiss J. P., Vizi E. S. (2001). Nitric oxide: a novel link between synaptic and nonsynaptic transmission. *Trends in Neurosciences*.

[59] Conti A., Miscusi M., Cardali S. (2007). Nitric oxide in the injured spinal cord: synthases cross-talk, oxidative stress and inflammation. *Brain Research Reviews*.

[60] Lundberg J. O., Weitzberg E., Gladwin M. T. (2008). The nitrate–nitrite–nitric oxide pathway in physiology and therapeutics. *Nature Reviews Drug Discovery*.

[61] Forstermann U. (2010). Nitric oxide and oxidative stress in vascular disease. *Pflügers Archiv - European Journal of Physiology*.

[62] Huang Z., Huang P. L., Ma J. (1996). Enlarged infarcts in endothelial nitric oxide synthase knockout mice are attenuated by nitro-L-arginine. *Journal of Cerebral Blood Flow & Metabolism*.

[63] Wei G., Dawson V. L., Zweier J. L. (1999). Role of neuronal and endothelial nitric oxide synthase in nitric oxide generation in the brain following cerebral ischemia. *Biochimica et Biophysica Acta (BBA) - Molecular Basis of Disease*.

[64] Samdani A. F., Dawson T. M., Dawson V. L. (1997). Nitric oxide synthase in models of focal ischemia. *Stroke*.

[65] Gursoy-Ozdemir Y., Bolay H., Saribas O., Dalkara T., Beckman J. S. (2000). Role of endothelial nitric oxide generation and peroxynitrite formation in reperfusion injury after focal cerebral ischemia. *Stroke*.

[66] Pacher P., Beckman J. S., Liaudet L. (2007). Nitric oxide and peroxynitrite in health and disease. *Physiological Reviews*.

[67] Gasche Y., Fujimura M., Morita-Fujimura Y. (1999). Early appearance of activated matrix metalloproteinase-9 after focal cerebral ischemia in mice: a possible role in blood—brain barrier dysfunction. *Journal of Cerebral Blood Flow & Metabolism*.

[68] Aoki T., Sumii T., Mori T., Wang X., Lo E. H. (2002). Blood-brain barrier disruption and matrix metalloproteinase-9 expression during reperfusion injury: mechanical versus embolic focal ischemia in spontaneously hypertensive rats. *Stroke*.

[69] Pfefferkorn T., Rosenberg G. A. (2003). Closure of the blood-brain barrier by matrix metalloproteinase inhibition reduces rtPA-mediated mortality in cerebral ischemia with delayed reperfusion. *Stroke*.

[70] Yang Y., Estrada E. Y., Thompson J. F., Liu W., Rosenberg G. A. (2007). Matrix metalloproteinase-mediated disruption of tight junction proteins in cerebral vessels is reversed by synthetic matrix metalloproteinase inhibitor in focal ischemia in rat. *Journal of Cerebral Blood Flow & Metabolism*.

[71] Rosenberg G. A. (2002). Matrix metalloproteinases in neuroinflammation. *Glia*.

[72] Lee C. Z., Xue Z., Zhu Y., Yang G. Y., Young W. L. (2007). Matrix metalloproteinase-9 inhibition attenuates vascular endothelial growth factor-induced intracerebral hemorrhage. *Stroke*.

[73] Rosenberg G. A., Yang Y. (2007). Vasogenic edema due to tight junction disruption by matrix metalloproteinases in cerebral ischemia. *Neurosurgical Focus*.

[74] Trimmer B. A., Aprille J. R., Dudzinski D. M. (2001). Nitric oxide and the control of firefly flashing. *Science*.

[75] Stamler J. S. (1994). Redox signaling: nitrosylation and related target interactions of nitric oxide. *Cell*.

[76] Matsushita K., Morrell C. N., Cambien B. (2003). Nitric oxide regulates exocytosis by S-nitrosylation of N-ethylmaleimide-sensitive factor. *Cell*.

[77] Nogawa S., Forster C., Zhang F., Nagayama M., Ross M. E., Iadecola C. (1998). Interaction between inducible nitric oxide synthase and cyclooxygenase-2 after cerebral ischemia. *Proceedings of the National Academy of Sciences of the United States of America*.

[78] Iadecola C., Gorelick P. B. (2005). The Janus face of cyclooxygenase-2 in ischemic stroke: shifting toward downstream targets. *Stroke*.

[79] Schopfer F. J., Baker P. R., Freeman B. A. (2003). NO-dependent protein nitration: a cell signaling event or an oxidative inflammatory response?. *Trends in Biochemical Sciences*.

[80] Kuhn D. M., Sakowski S. A., Sadidi M., Geddes T. J. (2004). Nitrotyrosine as a marker for peroxynitrite-induced neurotoxicity: the beginning or the end of the end of dopamine neurons?. *Journal of Neurochemistry*.

[81] Ischiropoulos H., Zhu L., Chen J. (1992). Peroxynitrite-mediated tyrosine nitration catalyzed by superoxide dismutase. *Archives of Biochemistry and Biophysics*.

[82] Lymar S. V., Hurst J. K. (1996). Carbon dioxide: physiological catalyst for peroxynitrite-mediated cellular damage or cellular protectant?. *Chemical Research in Toxicology*.

[83] Szabo C., Ischiropoulos H., Radi R. (2007). Peroxynitrite: biochemistry, pathophysiology and development of therapeutics. *Nature Reviews Drug Discovery*.

[84] Besson V. C. (2009). Drug targets for traumatic brain injury from poly(ADP-ribose)polymerase pathway modulation. *British Journal of Pharmacology*.

[85] Drapier J. C., Hibbs J. B. (1988). Differentiation of murine macrophages to express nonspecific cytotoxicity for tumor cells results in L-arginine-dependent inhibition of mitochondrial iron-sulfur enzymes in the macrophage effector cells. *The Journal of Immunology*.

[86] Lizasoain I., Moro M. A., Knowles R. G., Darley-Usmar V., Moncada S. (1996). Nitric oxide and peroxynitrite exert distinct effects on mitochondrial respiration which are differentially blocked by glutathione or glucose. *Biochemical Journal*.

[87] Sharpe M. A., Cooper C. E. (1998). Interaction of peroxynitrite with mitochondrial cytochrome oxidase. Catalytic production of nitric oxide and irreversible inhibition of enzyme activity. *Journal of Biological Chemistry*.

[88] Riobó N. A., Clementi E., Melani M. (2001). Nitric oxide inhibits mitochondrial NADH:ubiquinone reductase activity through peroxynitrite formation. *Biochemical Journal*.

[89] Aulak K. S., Koeck T., Crabb J. W., Stuehr D. J. (2004). Dynamics of protein nitration in cells and mitochondria. *American Journal of Physiology-Heart and Circulatory Physiology*.

[90] Guy R. A., Maguire G. F., Crandall I., Connelly P. W., Kain K. C. (2001). Characterization of peroxynitrite-oxidized low density lipoprotein binding to human CD36. *Atherosclerosis*.

[91] Rajagopalan S., Meng X. P., Ramasamy S., Harrison D. G., Galis Z. S. (1996). Reactive oxygen species produced by macrophage-derived foam cells regulate the activity of vascular matrix metalloproteinases in vitro. Implications for atherosclerotic plaque stability. *The Journal of Clinical Investigation*.

[92] Okamoto T., Akaike T., Sawa T., Miyamoto Y., van der Vliet A., Maeda H. (2001). Activation of matrix metalloproteinases by peroxynitrite-induced protein S-glutathiolation via disulfide S-oxide formation. *Journal of Biological Chemistry*.

[93] Viappiani S., Nicolescu A. C., Holt A. (2009). Activation and modulation of 72 kDa matrix metalloproteinase-2 by peroxynitrite and glutathione. *Biochemical Pharmacology*.

[94] Sandoval K. E., Witt K. A. (2008). Blood-brain barrier tight junction permeability and ischemic stroke. *Neurobiology of Disease*.

[95] Gursoy-Ozdemir Y., Can A., Dalkara T. (2004). Reperfusion-induced oxidative/nitrative injury to neurovascular unit after focal cerebral ischemia. *Stroke*.

[96] Guix F. X., Uribesalgo I., Coma M., Munoz F. J. (2005). The physiology and pathophysiology of nitric oxide in the brain. *Progress in Neurobiology*.

[97] Miller A. A., Drummond G. R., De Silva T. M. (2009). NADPH oxidase activity is higher in cerebral versus systemic arteries of four animal species: role of Nox2. *American Journal of Physiology-Heart and Circulatory Physiology*.

[98] Mathiisen T. M., Lehre K. P., Danbolt N. C., Ottersen O. P. (2010). The perivascular astroglial sheath provides a complete covering of the brain microvessels: an electron microscopic 3D reconstruction. *Glia*.

[99] Van der Goes A., Wouters D., Van Der Pol S. M. (2001). Reactive oxygen species enhance the migration of monocytes across the blood-brain barrier in vitro. *The FASEB Journal*.

[100] Lee H. S., Namkoong K., Kim D. H. (2004). Hydrogen peroxide-induced alterations of tight junction proteins in bovine brain microvascular endothelial cells. *Microvascular Research*.

[101] Adam-Vizi V. (2005). Production of reactive oxygen species in brain mitochondria: contribution by electron transport chain and non–electron transport chain sources. *Antioxidants & Redox Signaling*.

[102] Kahles T., Luedike P., Endres M. (2007). NADPH oxidase plays a central role in blood-brain barrier damage in experimental stroke. *Stroke*.

[103] Chrissobolis S., Faraci F. M. (2008). The role of oxidative stress and NADPH oxidase in cerebrovascular disease. *Trends in Molecular Medicine*.

[104] Lochhead J. J., McCaffrey G., Quigley C. E. (2010). Oxidative stress increases blood–brain barrier permeability and induces alterations in occludin during hypoxia–reoxygenation. *Journal of Cerebral Blood Flow & Metabolism*.

[105] Fraser P. A. (2011). The role of free radical generation in increasing cerebrovascular permeability. *Free Radical Biology & Medicine*.

[106] Lehner C., Gehwolf R., Tempfer H. (2011). Oxidative stress and blood–brain barrier dysfunction under particular consideration of matrix metalloproteinases. *Antioxidants & Redox Signaling*.

[107] Sumii T., Lo E. H. (2002). Involvement of matrix metalloproteinase in thrombolysis-associated hemorrhagic transformation after embolic focal ischemia in rats. *Stroke*.

[108] Wang X., Lee S. R., Arai K. (2003). Lipoprotein receptor–mediated induction of matrix metalloproteinase by tissue plasminogen activator. *Nature Medicine*.

[109] Zhao B. Q., Tejima E., Lo E. H. (2007). Neurovascular proteases in brain injury, hemorrhage and remodeling after stroke. *Stroke*.

[110] Ramos-Fernandez M., Bellolio M. F., Stead L. G. (2011). Matrix metalloproteinase-9 as a marker for acute ischemic stroke: a systematic review. *Journal of Stroke & Cerebrovascular Diseases*.

[111] Gugliucci A. (2003). Human plasminogen is highly susceptible to peroxynitrite inactivation. *Clinical Chemistry and Laboratory Medicine*.

[112] Nielsen V. G., Crow J. P., Zhou F., Parks D. A. (2004). Peroxynitrite inactivates tissue plasminogen activator. *Anesthesia & Analgesia*.

[113] Taffi R., Nanetti L., Mazzanti L. (2008). Plasma levels of nitric oxide and stroke outcome. *Journal of Neurology*.

[114] Rohn T. T., Hinds T. R., Vincenzi F. F. (1996). Inhibition of Ca^2+^-pump ATPase and the Na^+^/K^+^-pump ATPase by iron-generated free radicals: protection by 6,7-dimethyl-2,4-di-1-pyrrolidinyl-7*h*-pyrrolo[2,3-*d*]pyrimidine sulfate (U-89843D), a potent, novel, antioxidant/free radical scavenger. *Biochemical Pharmacology*.

[115] Kourie J. I. (1998). Interaction of reactive oxygen species with ion transport mechanisms. *American Journal of Physiology-Cell Physiology*.

[116] Sombati S., Coulter D. A., DeLorenzo R. J. (1991). Neurotoxic activation of glutamate receptors induces an extended neuronal depolarization in cultured hippocampal neurons. *Brain Research*.

[117] Przyklenk K., Bauer B., Ovize M., Kloner R. A., Whittaker P. (1993). Regional ischemic 'preconditioning' protects remote virgin myocardium from subsequent sustained coronary occlusion. *Circulation*.

[118] Veighey K., MacAllister R. J. (2012). Clinical applications of remote ischemic preconditioning. *Cardiology Research and Practice*.

[119] Schmidt M. R., Smerup M., Konstantinov I. E. (2007). Intermittent peripheral tissue ischemia during coronary ischemia reduces myocardial infarction through a K_ATP_-dependent mechanism: first demonstration of remote ischemic perconditioning. *American Journal of Physiology-Heart and Circulatory Physiology*.

[120] Hess D. C., Hoda M. N., Bhatia K. (2013). Remote limb perconditioning and postconditioning: will it translate into a promising treatment for acute stroke?. *Stroke*.

[121] Hahn C. D., Manlhiot C., Schmidt M. R., Nielsen T. T., Redington A. N. (2011). Remote ischemic per-conditioning: a novel therapy for acute stroke?. *Stroke*.

[122] Kerendi F., Kin H., Halkos M. E. (2005). Remote postconditioning. Brief renal ischemia and reperfusion applied before coronary artery reperfusion reduces myocardial infarct size via endogenous activation of adenosine receptors. *Basic Research in Cardiology*.

[123] Ren C., Gao X., Steinberg G. K., Zhao H. (2008). Limb remote-preconditioning protects against focal ischemia in rats and contradicts the dogma of therapeutic time windows for preconditioning. *Neuroscience*.

[124] Koch S., Katsnelson M., Dong C., Perez-Pinzon M. (2011). Remote ischemic limb preconditioning after subarachnoid hemorrhage: a phase Ib study of safety and feasibility. *Stroke*.

[125] Hu S., Dong H., Zhang H. (2012). Noninvasive limb remote ischemic preconditioning contributes neuroprotective effects via activation of adenosine A1 receptor and redox status after transient focal cerebral ischemia in rats. *Brain Research*.

[126] Hoda M. N., Siddiqui S., Herberg S. (2012). Remote ischemic perconditioning is effective alone and in combination with intravenous tissue-type plasminogen activator in murine model of embolic stroke. *Stroke*.

[127] Wei D., Ren C., Chen X., Zhao H. (2012). The chronic protective effects of limb remote preconditioning and the underlying mechanisms involved in inflammatory factors in rat stroke. *PLoS One*.

[128] Hougaard K. D., Hjort N., Zeidler D. (2014). Remote ischemic perconditioning as an adjunct therapy to thrombolysis in patients with acute ischemic stroke: a randomized trial. *Stroke*.

[129] Pan J., Li X., Peng Y. (2016). Remote ischemic conditioning for acute ischemic stroke: Dawn in the darkness. *Reviews in the Neurosciences*.

[130] Zeynalov E., Shah Z. A., Li R.-c., Doré S. (2009). Heme oxygenase 1 is associated with ischemic preconditioning-induced protection against brain ischemia. *Neurobiology of Disease*.

[131] Silachev D. N., Isaev N. K., Pevzner I. B. (2012). The mitochondria-targeted antioxidants and remote kidney preconditioning ameliorate brain damage through kidney-to-brain cross-talk. *PLoS One*.

[132] Pignataro G., Esposito E., Sirabella R. (2013). nNOS and p-ERK involvement in the neuroprotection exerted by remote postconditioning in rats subjected to transient middle cerebral artery occlusion. *Neurobiology of Disease*.

[133] Lu X. M., Zhang G. X., Yu Y. Q. (2009). The opposite roles of nNOS in cardiac ischemia–reperfusion-induced injury and in ischemia preconditioning-induced cardioprotection in mice. *The Journal of Physiological Sciences*.

[134] Iadecola C., Kahles T., Gallo E. F., Anrather J. (2011). Neurovascular protection by ischaemic tolerance: role of nitric oxide. *The Journal of Physiology*.

[135] Peng B., Guo Q.-l., He Z.-j. (2012). Remote ischemic postconditioning protects the brain from global cerebral ischemia/reperfusion injury by up-regulating endothelial nitric oxide synthase through the PI3K/Akt pathway. *Brain Research*.

[136] Chen G., Ye X., Zhang J. (2016). Limb remote ischemic postconditioning reduces ischemia-reperfusion injury by inhibiting NADPH oxidase activation and MyD88-TRAF6-P38MAP-kinase pathway of neutrophils. *International Journal of Molecular Sciences*.

[137] Konstantinov I. E., Arab S., Kharbanda R. K. (2004). The remote ischemic preconditioning stimulus modifies inflammatory gene expression in humans. *Physiological Genomics*.

[138] Henninger N., Fisher M. (2007). Stimulating circle of Willis nerve fibers preserves the diffusion-perfusion mismatch in experimental stroke. *Stroke*.

[139] Sun Z., Baker W., Hiraki T., Greenberg J. H. (2012). The effect of right vagus nerve stimulation on focal cerebral ischemia: an experimental study in the rat. *Brain Stimulation*.

[140] Han Z., Liu X., Luo Y., Ji X. (2015). Therapeutic hypothermia for stroke: where to go?. *Experimental Neurology*.

[141] Lyden P. D., Krieger D., Yenari M., Dietrich W. D. (2006). Therapeutic hypothermia for acute stroke. *International Journal of Stroke*.

[142] Wang Q., Li A. L., Zhi D. S., Huang H. L. (2007). Effect of mild hypothermia on glucose metabolism and glycerol of brain tissue in patients with severe traumatic brain injury. *Chinese Journal of Traumatology*.

[143] Kim J. Y., Kim N., Yenari M. A., Chang W. (2011). Mild hypothermia suppresses calcium-sensing receptor (CaSR) induction following forebrain ischemia while increasing GABA-B receptor 1 (GABA-B-R1) expression. *Translational Stroke Research*.

[144] Wenisch C., Narzt E., Sessler D. I. (1996). Mild intraoperative hypothermia reduces production of reactive oxygen intermediates by polymorphonuclear leukocytes. *Anesthesia and Analgesia*.

[145] Lei B., Adachi N., Arai T. (1997). The effect of hypothermia on H_2_O_2_ production during ischemia and reperfusion: a microdialysis study in the gerbil hippocampus. *Neuroscience Letters*.

[146] Horiguchi T., Shimizu K., Ogino M., Suga S., Inamasu J., Kawase T. (2003). Postischemic hypothermia inhibits the generation of hydroxyl radical following transient forebrain ischemia in rats. *Journal of Neurotrauma*.

[147] McManus T., Sadgrove M., Pringle A. K., Chad J. E., Sundstrom L. E. (2004). Intraischaemic hypothermia reduces free radical production and protects against ischaemic insults in cultured hippocampal slices. *Journal of Neurochemistry*.

[148] Ji X., Luo Y., Ling F. (2007). Mild hypothermia diminishes oxidative DNA damage and pro-death signaling events after cerebral ischemia: a mechanism for neuroprotection. *Frontiers in Bioscience*.

[149] Phanithi P. B., Yoshida Y., Santana A., Su M., Kawamura S., Yasui N. (2000). Mild hypothermia mitigates post-ischemic neuronal death following focal cerebral ischemia in rat brain: immunohistochemical study of Fas, caspase-3 and TUNEL. *Neuropathology*.

[150] Han H. S., Qiao Y., Karabiyikoglu M., Giffard R. G., Yenari M. A. (2002). Influence of mild hypothermia on inducible nitric oxide synthase expression and reactive nitrogen production in experimental stroke and inflammation. *The Journal of Neuroscience*.

[151] Deng H., Han H. S., Cheng D., Sun G. H., Yenari M. A. (2003). Mild hypothermia inhibits inflammation after experimental stroke and brain inflammation. *Stroke*.

[152] Liu L., Kim J. Y., Koike M. A. (2008). FasL shedding is reduced by hypothermia in experimental stroke. *Journal of Neurochemistry*.

[153] Xiong M., Yang Y., Chen G. Q., Zhou W. H. (2009). Post-ischemic hypothermia for 24h in P7 rats rescues hippocampal neuron: association with decreased astrocyte activation and inflammatory cytokine expression. *Brain Research Bulletin*.

[154] Liu X., Wang M., Chen H. (2013). Hypothermia protects the brain from transient global ischemia/reperfusion by attenuating endoplasmic reticulum response-induced apoptosis through CHOP. *PLoS One*.

[155] Gu L. J., Xiong X. X., Ito T. (2014). Moderate hypothermia inhibits brain inflammation and attenuates stroke-induced immunodepression in rats. *CNS Neuroscience & Therapeutics*.

[156] Lee J. E., Yoon Y. J., Moseley M. E., Yenari M. A. (2005). Reduction in levels of matrix metalloproteinases and increased expression of tissue inhibitor of metalloproteinase—2 in response to mild hypothermia therapy in experimental stroke. *Journal of Neurosurgery*.

[157] Gao D., Ding F., Lei G. (2015). Effects of focal mild hypothermia on thrombin-induced brain edema formation and the expression of protease activated receptor-1, matrix metalloproteinase-9 and aquaporin 4 in rats. *Molecular Medicine Reports*.

[158] Yenari M. A., Palmer J. T., Bracci P. M., Steinberg G. K. (1995). Thrombolysis with tissue plasminogen activator (tPA) is temperature dependent. *Thrombosis Research*.

[159] Kallmunzer B., Schwab S., Kollmar R. (2012). Mild hypothermia of 34°C reduces side effects of rt-PA treatment after thromboembolic stroke in rats. *Experimental & Translational Stroke Medicine*.

[160] Zarisfi M., Allahtavakoli F., Hassanipour M. (2017). Transient brain hypothermia reduces the reperfusion injury of delayed tissue plasminogen activator and extends its therapeutic time window in a focal embolic stroke model. *Brain Research Bulletin*.

[161] Kollmar R., Henninger N., Bardutzky J., Schellinger P. D., Schäbitz W.-R., Schwab S. (2004). Combination therapy of moderate hypothermia and thrombolysis in experimental thromboembolic stroke—an MRI study. *Experimental Neurology*.

[162] Hemmen T. M., Raman R., Guluma K. Z. (2010). Intravenous thrombolysis plus hypothermia for acute treatment of ischemic stroke (ICTuS-L): final results. *Stroke*.

[163] Bi M., Ma Q., Zhang S. (2011). Local mild hypothermia with thrombolysis for acute ischemic stroke within a 6-h window. *Clinical Neurology and Neurosurgery*.

[164] Piironen K., Tiainen M., Mustanoja S. (2014). Mild hypothermia after intravenous thrombolysis in patients with acute stroke: a randomized controlled trial. *Stroke*.

[165] Hong J. M., Lee J. S., Song H. J., Jeong H. S., Choi H. A., Lee K. (2014). Therapeutic hypothermia after recanalization in patients with acute ischemic stroke. *Stroke*.

[166] Ma L. L., Song L., Yu X. D., Yu T. X., Liang H., Qiu J. X. (2017). The clinical study on the treatment for acute cerebral infarction by intra-arterial thrombolysis combined with mild hypothermia. *European Review for Medical and Pharmacological Sciences*.

[167] Abe K., Yuki S., Kogure K. (1988). Strong attenuation of ischemic and postischemic brain edema in rats by a novel free radical scavenger. *Stroke*.

[168] Morita M., Naito Y., Yoshikawa T., Niki E. (2016). Inhibition of plasma lipid oxidation induced by peroxyl radicals, peroxynitrite, hypochlorite, 15-lipoxygenase, and singlet oxygen by clinical drugs. *Bioorganic & Medicinal Chemistry Letters*.

[169] Yamamoto Y. (2017). Plasma marker of tissue oxidative damage and edaravone as a scavenger drug against peroxyl radicals and peroxynitrite. *Journal of Clinical Biochemistry and Nutrition*.

[170] Yamashita T., Shoge M., Oda E. (2006). The free-radical scavenger, edaravone, augments NO release from vascular cells and platelets after laser-induced, acute endothelial injury *in vivo*. *Platelets*.

[171] Yagi K., Kitazato K. T., Uno M. (2009). Edaravone, a free radical scavenger, inhibits MMP-9–related brain hemorrhage in rats treated with tissue plasminogen activator. *Stroke*.

[172] Kano T., Harada T., Katayama Y. (2005). Attenuation of extravasation of tissue plasminogen activator by the free radical scavenger, edaravone: evaluation in a rat thromboembolic stroke model. *Neurological Research*.

[173] Yamashita T., Sato T., Sakamoto K., Ishii H., Yamamoto J. (2015). The free-radical scavenger edaravone accelerates thrombolysis with alteplase in an experimental thrombosis model. *Thrombosis Research*.

[174] Kimura K., Aoki J., Sakamoto Y. (2012). Administration of edaravone, a free radical scavenger, during t-PA infusion can enhance early recanalization in acute stroke patients — a preliminary study. *Journal of the Neurological Sciences*.

[175] Wada T., Yasunaga H., Inokuchi R. (2014). Effects of edaravone on early outcomes in acute ischemic stroke patients treated with recombinant tissue plasminogen activator. *Journal of the Neurological Sciences*.

[176] Yamaguchi T., Awano H., Matsuda H., Tanahashi N. (2017). Edaravone with and without .6 mg/kg alteplase within 4.5 hours after ischemic stroke: a prospective cohort study (PROTECT4.5). *Journal of Stroke & Cerebrovascular Diseases*.

[177] Kono S., Deguchi K., Morimoto N. (2013). Intravenous thrombolysis with neuroprotective therapy by edaravone for ischemic stroke patients older than 80 years of age. *Journal of Stroke & Cerebrovascular Diseases*.

[178] Squadrito G. L., Cueto R., Splenser A. E. (2000). Reaction of uric acid with peroxynitrite and implications for the mechanism of neuroprotection by uric acid. *Archives of Biochemistry and Biophysics*.

[179] Hink H. U., Santanam N., Dikalov S., McCann L., Nguyen A. D. (2002). Peroxidase properties of extracellular superoxide dismutase: role of uric acid in modulating in vivo activity. *Arteriosclerosis, Thrombosis, and Vascular Biology*.

[180] Logallo N., Naess H., Idicula T. T., Brogger J., Waje-Andreassen U., Thomassen L. (2011). Serum uri acid: neuroprotection in thrombolysis. The Bergen NORSTROKE study. *BMC Neurology*.

[181] Amaro S., Urra X., Gomez-Choco M. (2011). Uric acid levels are relevant in patients with stroke treated with thrombolysis. *Stroke*.

[182] Liu X., Liu M., Chen M., Ge Q. M., Pan S. M. (2015). Serum uric acid is neuroprotective in Chinese patients with acute ischemic stroke treated with intravenous recombinant tissue plasminogen activator. *Journal of Stroke & Cerebrovascular Diseases*.

[183] Lee S. H., Heo S. H., Kim J. H. (2014). Effects of uric acid levels on outcome in severe ischemic stroke patients treated with intravenous recombinant tissue plasminogen activator. *European Neurology*.

[184] Romanos E., Planas A. M., Amaro S., Chamorro A. (2007). Uric acid reduces brain damage and improves the benefits of rt-PA in a rat model of thromboembolic stroke. *Journal of Cerebral Blood Flow & Metabolism*.

[185] Chamorro A., Amaro S., Castellanos M. (2017). Uric acid therapy improves the outcomes of stroke patients treated with intravenous tissue plasminogen activator and mechanical thrombectomy. *International Journal of Stroke*.

[186] Llull L., Laredo C., Renú A. (2015). Uric acid therapy improves clinical outcome in women with acute ischemic stroke. *Stroke*.

[187] Zhang X., Huang Z. C., Lu T. S., You S. J., Cao Y. J., Liu C. F. (2016). Prognostic significance of uric acid levels in ischemic stroke patients. *Neurotoxicity Research*.

[188] Irie F., Kamouchi M., Hata J. (2015). Sex differences in short-term outcomes after acute ischemic stroke: the fukuoka stroke registry. *Stroke*.

[189] Liu F., Li Z., Li J., Siegel C., Yuan R., McCullough L. D. (2009). Sex differences in caspase activation after stroke. *Stroke*.

[190] Trovarelli G., De Medio G. E., Dorman R. V., Piccinin G. L., Horrocks L. A., Porcellati G. (1981). Effect of cytidine diphosphate choline (CDP-choline) on ischemia-induced alterations of brain lipid in the gerbil. *Neurochemical Research*.

[191] Weiss G. B. (1995). Metabolism and actions of cdpcholine as an endogenous compound and administered exogenously as citicoline. *Life Sciences*.

[192] Krupinski J., Ferrer I., Barrachina M., Secades J. J., Mercadal J., Lozano R. (2002). CDP-choline reduces pro-caspase and cleaved caspase-3 expression, nuclear DNA fragmentation, and specific PARP-cleaved products of caspase activation following middle cerebral artery occlusion in the rat. *Neuropharmacology*.

[193] Secades J. J., Lorenzo J. L. (2006). Citicoline: pharmacological and clinical review, 2006 update. *Methods and Findings in Experimental and Clinical Pharmacology*.

[194] Martynov M. Y., Gusev E. I. (2015). Current knowledge on the neuroprotective and neuroregenerative properties of citicoline in acute ischemic stroke. *Journal of Experimental Pharmacology*.

[195] Katsuki H., Okuda S. (1995). Arachidonic acid as a neurotoxic and neurotrophic substance. *Progress in Neurobiology*.

[196] Agut J., Lopez G. C. I., Ortiz J. A., Wurtman R. J. (1993). Oral cytidine 5′-diphosphate choline administration to rats increases brain phospholipid levels. *Annals of the New York Academy of Sciences*.

[197] Adibhatla R. M., Hatcher J. F. (2002). Citicoline mechanisms and clinical efficacy in cerebral ischemia. *Journal of Neuroscience Research*.

[198] Adibhatla R. M., Hatcher J. F., Dempsey R. J. (2002). Citicoline: neuroprotective mechanisms in cerebral ischemia. *Journal of Neurochemistry*.

[199] Adibhatla R. M., Hatcher J. F. (2003). Citicoline decreases phospholipase A2 stimulation and hydroxyl radical generation in transient cerebral ischemia. *Journal of Neuroscience Research*.

[200] Serra I., Alberghina M., Viola M., Mistretta A., Giuffrida A. M. (1981). Effect of CDP-choline on the biosynthesis of nucleic acids and proteins in brain regions during hypoxia. *Neurochemical Research*.

[201] Secades J. J. (2011). Citicoline: pharmacological and clinical review, 2010 update. *Revista de Neurologia*.

[202] Rao A. M., Hatcher J. F., Dempsey R. J. (1999). CDP-choline: neuroprotection in transient forebrain ischemia of gerbils. *Journal of Neuroscience Research*.

[203] Oshitari T., Fujimoto N., Adachi-Usami E. (2002). Citicoline has a protective effect on damaged retinal ganglion cells in mouse culture retina. *NeuroReport*.

[204] Barrachina M., Secades J., Lozano R., Gómez-Santos C., Ambrosio S., Ferrer I. (2002). Citicoline increases glutathione redox ratio and reduces caspase-3 activation and cell death in staurosporine-treated SH-SY5Y human neuroblastoma cells. *Brain Research*.

[205] Mir C., Clotet J., Aledo R. (2003). CDP-choline prevents glutamate-mediated cell death in cerebellar granule neurons. *Journal of Molecular Neuroscience*.

[206] Matyja E., Taraszewska A., Naganska E., Grieb P., Rafalowska J. (2008). CDP-choline protects motor neurons against apoptotic changes in a model of chronic glutamate excitotoxicity in vitro. *Folia Neuropathologica*.

[207] Clark W. M., Williams B. J., Selzer K. A., Zweifler R. M., Sabounjian L. A., Gammans R. E. (1999). A randomized efficacy trial of citicoline in patients with acute ischemic stroke. *Stroke*.

[208] Clark W. M., Wechsler L. R., Sabounjian L. A., Schwiderski U. E. (2001). A phase III randomized efficacy trial of 2000 mg citicoline in acute ischemic stroke patients. *Neurology*.

[209] Davalos A., Castillo J., Alvarez-Sabin J. (2002). Oral citicoline in acute ischemic stroke: an individual patient data pooling analysis of clinical trials. *Stroke*.

[210] Andersen M., Overgaard K., Meden P., Boysen G., Choi S. C. (1999). Effects of citicoline combined with thrombolytic therapy in a rat embolic stroke model. *Stroke*.

[211] Alonso de Lecinana M., Gutierrez M., Roda J. M., Carceller F., Diez-Tejedor E. (2006). Effect of combined therapy with thrombolysis and citicoline in a rat model of embolic stroke. *Journal of the Neurological Sciences*.

[212] Dávalos A., Alvarez-Sabín J., Castillo J. (2012). Citicoline in the treatment of acute ischaemic stroke: an international, randomised, multicentre, placebo-controlled study (ICTUS trial). *The Lancet*.

